# Cost-effectiveness analysis of sedentary behaviour interventions in offices to reduce sitting time in Australian desk-based workers: A modelling study

**DOI:** 10.1371/journal.pone.0287710

**Published:** 2023-06-29

**Authors:** Phuong Nguyen, Jaithri Ananthapavan, Lan Gao, David W. Dunstan, Marj Moodie

**Affiliations:** 1 Deakin Health Economics, School of Health and Social Development, Institute for Health Transformation, Deakin University, Geelong, Australia; 2 Global Obesity Centre, School of Health and Social Development, Institute for Health Transformation, Deakin University, Geelong, Australia; 3 School of Biomedical Sciences and Pharmacy, The University of Newcastle, Callaghan, NSW, Australia; 4 Baker Heart and Diabetes Institute, Melbourne, Australia; 5 Mary MacKillop Institute for Health Research, Australian Catholic University, Melbourne, Victoria, Australia; 6 Institute for Physical Activity and Nutrition, Faculty of Health, Deakin University, Geelong, Vitoria, Australia; University of Huddersfield, UNITED KINGDOM

## Abstract

**Objectives:**

Sedentary behaviour (SB) is associated with increased incidence of chronic diseases such as type 2 diabetes (T2D), cardiovascular disease, cancers, and premature mortality. SB interventions in workplaces are effective in reducing sitting time. Previous economic evaluations have not specifically used changes in sitting time to estimate the long-term impact of SB on chronic disease-related health and cost outcomes. This research evaluated the cost-effectiveness of three hypothetical SB interventions: behavioural (BI), environmental (EI) and multi-component intervention (MI), implemented in the Australian context, using a newly developed epidemiological model that estimates the impact of SB as a risk factor on long-term population health and associated cost outcomes.

**Method:**

Pathway analysis was used to identify the resource items associated with implementing each of the three interventions using a limited societal perspective (included costs: health sector, individuals and industry; excluded costs: productivity). The effectiveness of the modelled interventions in reducing daily sitting time (informed by published meta-analyses) was modelled for the Australian working population aged 20–65 years. A multi-cohort Markov model was developed to simulate the 2019 Australian population and estimate the incidence, prevalence and mortality of five diseases causally related to excessive sitting time, over the life course. Monte-Carlo simulations were used to calculate each intervention’s mean incremental costs and benefits (quantified as health adjusted life years HALYs) compared to a do-nothing comparator.

**Results:**

When implemented at the national level, the interventions were estimated to reach 1,018 organisations with 1,619,239 employees. The estimated incremental cost of SB interventions was A$159M (BI), A$688M (EI) and A$438M (MI) over a year. Incremental health-adjusted life years (HALYs) gained by BI, EI and MI were 604, 919 and 349, respectively. The mean ICER for BI was A$251,863 per HALY gained, A$737,307 for EI and A$1,250,426 for MI. Only BI had any probability (2%) of being cost-effective at a willingness-to-pay threshold of A$50,000 per HALY gained from a societal perspective.

**Conclusion:**

SB interventions are not cost-effective when a reduction in sitting time is the outcome measure of interest. The cost-effectiveness results are heavily driven by the cost of the sit-stand desks and the small HALYs gained from reducing sitting time. Future research should focus on capturing non-health-benefits of these interventions, such as productivity, work satisfaction, and other health benefits: metabolic, physical, and musculoskeletal outcomes. Importantly, the health benefits of simultaneously reducing sitting time and increasing standing time for such interventions should be captured with the joint effects of these risk factors appropriately considered.

## Introduction

Sedentary behaviour (SB), i.e. excessive sitting, has become an established behavioural risk factor and is independently associated with chronic diseases such as type 2 diabetes (T2D), cardiovascular disease and cancers and is associated with premature mortality [1–3]. Substantial evidence also suggests an association between excessive and/or prolonged sitting time and lost productivity during paid work (i.e. absenteeism due sickness and presenteeism) [4, 5]. In the short term, reduction in excessive sitting time and the introduction of regular active breaks are likely to improve musculoskeletal discomfort and the health of office workers [6–8].

The Australian National Health Survey 2018 reported that 44% of the adult population described their time at work as ‘mostly sitting’ [9]. Studies in other high-income countries demonstrated that urban desk-based working populations engaged in SB for excessive and prolonged periods of time, spending an average of 77% of their awake time sitting in the office uninterrupted [10, 11], driving to work, and were also sedentary during leisure time [12, 13]. A study by Ding et al. [14] showed that, in Australia, the average total time taken to drive to work was approximately 84 mins/day, with an estimated 60% of the population driving to work daily. Sedentary behaviour is, therefore, a public health concern, and workplaces have been identified as a priority setting for interventions to reduce excessive and prolonged sitting [15, 16].

Sedentary behaviour interventions in workplaces are reported to be effective in reducing sitting time [17–21]. Systematic reviews with meta-analyses have investigated the effectiveness of different SB interventions in the office setting, such as behavioural interventions (BIs), environmental interventions (EIs), or multi-component interventions (MI) that combine both behavioural and environmental components [18, 19]. All three types of interventions are reported to successfully reduce occupational sitting time in office-based workers with estimated reductions in sitting time ranging from 30 minutes per day to 100 minutes per 8-hour work day [17].

To date, only three economic evaluations of SB interventions have been conducted and have shown varied results [22]. All studies reported on multi-component SB interventions. One study reported the intervention approach as cost-effective compared with no intervention (Stand-Up Victoria trial in Australia [23]); one was less costly, but also less effective when compared to active controls (Dynamic Work trial in the Netherlands [24]), and the third one reported a positive benefit-cost ratio (SMArt Work trial in the UK [25]). The methods and inputs to estimate the change in long-term health and cost outcomes also varied. One used increases in standing time rather than changes in SB (Stand-Up Victoria [23]), one directly estimated changes in health related quality of life resulting from the intervention (Dynamic Work [24]), and one estimated the change in productivity (SMArt Work [25]). None of the above evaluations has used reduction in sitting time as a risk factor to model the long-term impact of SB interventions related health and cost outcomes. Meanwhile, SB is recognised as a distinct risk factor from physical inactivity [1, 26], and associated with different chronic diseases [2, 3]. For instance, physical activity lowers the incidence of breast, colon, endometrium, esophagus, kidney, stomach, and lung cancers; heart disease, stroke, hypertension, and T2D [2]. SB is associated with T2D, stroke (but not heart disease), and fewer types of cancers (breast cancer, colon cancer, endometrial cancer) [2, 27]. The health impact from reduction in sitting time is consequently different to increase in physical activity. Moreover, the current evidence indicates that economic evaluations have focused solely on MIs, yet, while other SB interventions (BIs, EIs) were reported to produce similar effect sizes; none of these interventions have been evaluated within economic analyses.

To address this gap in evidence, we applied a newly developed model [27] that uses reduction in sitting time as the risk factor to evaluate the long term cost and health impacts of SB interventions. Specifically, we evaluated three hypothetical SB interventions: BI, EI and MI. The results of this current study are expected to provide new evidence to inform investment decisions in SB interventions in the workplace by policymakers and other stakeholders in Australia.

## Method

### Definition of the interventions to be modelled

**Fig 1:**
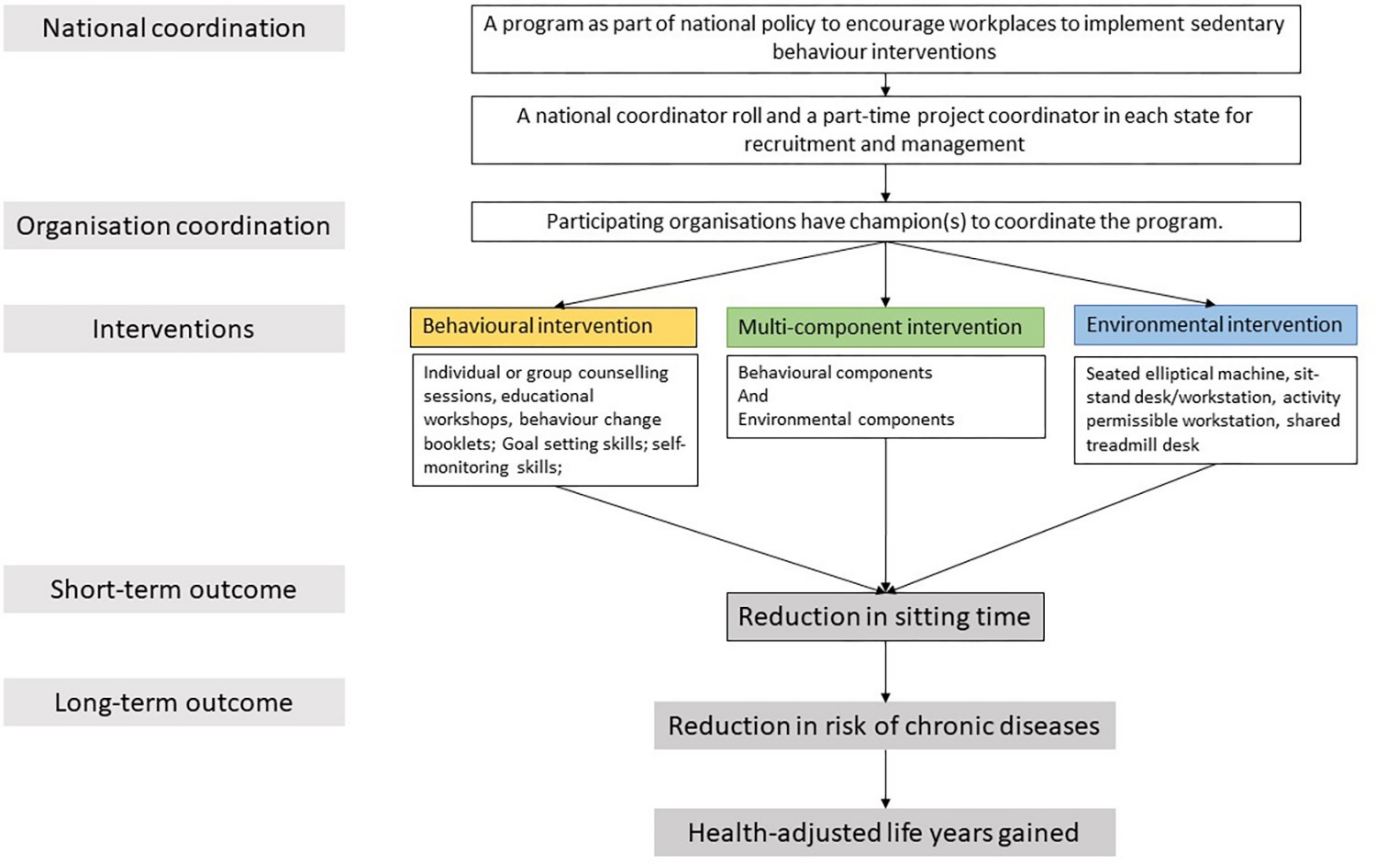
Intervention pathway.

It was assumed that these initiatives were a part of a national policy to encourage workplaces to implement SB interventions relevant to their context. Based on the two most current systematic reviews by Blackburn et al [19] and Shrestha et al. [21], hypothetical workplace SB interventions appropriate for the Australian setting were defined and costed. SB interventions of interest were categorised into three distinct types. BI employed behaviour change techniques targeted at individuals; this intervention includes individual or group counselling sessions, educational workshops, and educational emails, and follow-up phone calls on progress, goal setting, goals adjustment etc. [18, 19, 21] EI facilitated physical changes in the workplace environment, i.e. replacing traditional sit desks with sit-stand desks, or active workstations such as treadmill desk, under desk elliptical pedal [18, 19, 21]. MI combined both behavioural and environmental components [18, 19]. Fig 1 below showed the intervention pathway.

Whilst a feature of SB interventions includes organizational involvement in determining the appropriate number and nature of strategies suitable for individual organization contexts, for the purpose of this study, we developed a program based on different studies, which were presentative for SB interventions implementing in different organisations. The intervention was defined as a policy initiative administered across Australia by a Federal agency. In Australia, suitable agencies include Safe Work Australia—a government statutory agency to develop national policy to improve work health and safety [28]; or Comcare—the national work health and safety, and workers’ compensation authority [29].

The SB interventions targeted Australian desk-based office workers. Based on data of a similar intervention, the BeUpstanding trial [30], it was assumed that 0.37% of Australian businesses with more than five employees would participate in the program [31]. Uptake of the various interventions by individual workers was based on a systematic review by Blackburn et al [19], who took into account those who were not eligible to use a sit-to-stand desk or unable to stand for prolonged periods, such as people with established cardiovascular disease, acute illness or injury, epilepsy, pregnancy and dizziness, cognitive impairments, or using medications contraindicated with physical activity [32, 33]. Intervention strategies and resource use were synthesised from trials included in the review by Blackburn et al. [19] and are reported in Table 1 below. The champion refers to the contact person within each organisation, who advocated for strategies within organisations and co-ordinated the implementation of the agreed strategies (except for EI). In a large organisation, a champion could be the organisation’s health and safety officer, while in smaller organisations, this role could be undertaken by a person from Human resource or an administration role.

**Table 1:** Interventions strategies and resource use.

Policy intervention to encourage workplaces to implement sedentary behaviour interventions in office workplaces to reduce occupational sitting across Australia. Administered by a Federal Government agency. Resource use for policy administration: 1 full time project manager (to coordinate the program at national level); 1 part-time (0.5 full-time equivalent) project officer in each state and territory [23]			
**Targeted organization**	Australian business in 2019 with at least 5 employees: 273,829 businesses (ABS Counts of Australian Businesses, Table 13a [34])		
**Organization Uptake**	0.37% of total business (≥5 employees) in Australia in 2019		
**Number of champions**	Ratio 1.27 champions per organisation (291 champions representing 230 organisations) [35]		
**Intervention Type**	**Behavioural intervention**	**Environmental intervention**	**Multi-component intervention**
**Individual uptake**	39% (2% [36] to 77% [37])	57% (24% [38] to 75% [39])	32% (10% [40] to 59% [41])
**Organisation engagement**			
Meeting between project manager and organisation managers to introduce the program	One-off meeting; duration range 45 minutes [42] to 3 hours [43]		
**Capacity building for champion/team leader**			
Training of champions by occupational health professional: educational information about SB and health, behaviour change etc.	2 hours [36]	Not applicable	1 hour [44]- 3 hours [43]
**Counselling for behaviour change**			
Group counselling regarding excessive sitting and its impact on health; sharing experiences & tips among participants	6 sessions (range: 2 [36] -10 [37]); duration 1 hour (range: 0.75–1.5)		
Individual face-to-face counselling between occupational health professional and participants	3 sessions (range: 0–5 [36]); duration: 20–30 minutes [36]		4 sessions (range: 1 [45, 46]- 8 [43]); duration: 30 minutes [43, 45] (range: 10 [41]– 45 [43])
Group emails	3 emails (range: 0–5 [37]); duration 7 minutes (range: 5–10)		4 emails (range: 3–7 [44, 45]); duration 7 minutes (range: 5–10)
Individual phone calls: follow-up on progress, goal setting, goals adjustment	Not applicable		5 phone calls (range: 3 [45] - 6 [46]); duration: 10 minutes [45, 46] (range: 5–15)
**Equipment**			
Shared Active workstations: treadmill desk/ under desk elliptical pedal		27% of targeted population receive shared active workstations; ratio 4 employees per 1 active workstation [24]	
Sit-stand-desk (height-adjustable workstation or sit-stand platforms)		73% or targeted population receive sit-stand desk. It is assumed that half of this group receive sit-stand work stations and half receive sit-stand platforms [24]	
Instruction to use the equipment; posture while standing and sitting.		Video instruction 10 minutes (range 5–15)	

Note: Pert distributions were used to quantify the uncertainty around resource use estimates. Pert distribution is a continuous probability distributions defined by the mean, minimum and maximum values.

### Assessing costs of interventions

Pathway analysis was used to identify the resource items associated with the implementation of each the three intervention approaches (BI, EI, and MI). It was assumed that each intervention was operating in steady state (running at its full effectiveness potential); so, whilst the costs of organisation engagement were incorporated, research and development costs of individual strategies were excluded. Total costs comprised the cost of intervention delivery. Cost of adverse events attributable to the intervention were excluded due to the limitations in the available data [18, 19]. No costs were assumed in the usual practice control group. The participant time for engaging with the intervention was work hours and therefore the cost was accrued by businesses. Based on the assumptions made in Stand Up Victoria [23], we assumed that overhead management required one full time project manager for a national co-ordination level and one part-time project officer in each state and territory.

Unit costs and resource use for implementing the three types of SB interventions were compiled from a variety of sources including process data from trials and other published SB interventions. Equipment costs were not annuitized. Personnel time costs were valued using hourly wage rates for the Australian Bureau of statistics and including 14% salary on-costs [47]. Costs were adjusted to 2019 prices using the Consumer Price Index reported by the Australian Bureau of Statistics [48], where necessary. A summary of key unit costs, resource use and associated assumptions are reported in Table 2.

**Table 2:** Unit costs and uncertainty distribution.

Item	Unit cost (AUD)	Distribution	Assumptions and source
**Salary wage costs**			
Project manager (annual wage)	$113,251 (range: $103,764 to $118,926)	Pert	Mid-point EL1.4 (EL1.1 to EL1.7)
			The Australian Public Service Enterprise Agreement 2018–21 [49]
Project officer (annual wage)	$85,235 (range: $74,866 to $92,535)	Pert	Mid-point APS6.1 (APS5.1 to APS6.3) [49]
Occupational health and safety professionals (weekly earning)	$1,661.70 (SE 53.3)	Gamma	Occupational and Environmental Health professionals–ABS weekly earning 2014 (Table 1) [50]
Business manager (hourly earnings)	$57.00 (RSE 4%)	Gamma	ABS Average hourly earnings 2019 [51]
Champion and intervention participant (hourly earnings)	$32.50 (RSE 0.001%)	Gamma	ABS Average hourly earnings 2019
			Applied for champion and participant time [51]
**Equipment–for individual use: 73% of participants received individual equipment**			
Sit-stand workstation	$703.00 to $960.00	Pert	Ergotron Single/Duo Workfit station price 2021 [52, 53]
Sit-stand platform	$550.00 to $835.00	Pert	VARIDesk platform price 2021 [53]
Assembly and/or delivery fee	$99 to $240	Pert	Cost per item; price 2021 [53, 54]
**Equipment for share: 27% of participants received shared equipment, ratio 1:4**			
Treadmill workstation	$1,279 to $1,749	Pert	Lifespan Fitness price 2021 [55]
Assembly and fee	$100		Lifespan Fitness price 2021 [56]
Under desk elliptical pedal	$269 to $349.00		Free delivery, no assembly fee required. Price 2021 [57]
**Materials**			
Information booklet	$1.22 ($0.98 to $1.46)	Pert	Officeworks price for booklet printing price 2021 [58]
Diary/ activity tracker	$5.50 ($4.50 to $6.50)	Pert	Officeworks price for diaries/planner price 2021 [59]
**Website maintenance**			
Annual fee, incl. host, general maintenance and Cloud database	$3,745 to $9,156	Pert	E-market webpage [60]

Note: ABS: Australian Bureau of Statistic; APS: Australia Public Service; EL: Executive level; RSE: relative standard error; $: Australian dollar; Prices were inflated or deflated to 2019 values where needed using the consumer price index [48].

### Assessing interventions effectiveness

Intervention effectiveness was based on the reduction in sitting time by type of intervention reported in the most recent systematic review by Blackburn et al 2021 [19]. Meta-analyses by Blackburn and co-authors reported [19], compared to do-nothing (usual working environment) controls, a mean reduction in daily sitting time of 60.57 minutes (95% confidence interval (CI): 34.46 to 86.67) for behavioural interventions; 64.05 minutes (95% CI: 23.46 to 104.64) for environmental interventions; and 42.63 minutes daily (95% CI: 7.63 to 77.62) for multi-component interventions.

### Health outcomes modelling

The impact of SB interventions on selected chronic diseases was modelled to estimate long-term health and cost outcomes. The effectiveness of the interventions in reducing daily sitting time (informed by the meta-analysis [19]) was modelled for the Australian working population age 20–65 years. Given the limited evidence on maintenance of intervention effects, it was assumed that the changes in sitting time as a result of the intervention would decay by 10% annually in the intervention population [23].

The ACE-Obesity Policy model [61], a proportional, multi-state life table Markov cohort model, was enhanced to estimate the short and long-term health outcomes resulting from changes in sitting time. The details of the model have been previously published [27]. All information regarding the scope of the model, disease transition and other model variables (population, mean sitting time by categories, disease relative risk etc.) can be freely accessed at https://doi.org/10.1186/s12966-022-01276-2. The model is briefly described here.

The model simulates the 2019 Australian population and estimates the incidence, prevalence and mortality of five diseases causally related to excessive sitting time over the life course (T2D, stroke, and endometrial, breast and colorectal cancers). Each of these diseases was modelled as transitions between four health states (healthy, diseased, dead due to disease, and dead from other causes) over one year cycles. Transitions between states were determined by incidence (calculated using potential impact fractions (PIF) [62] based on relative risk of disease), prevalence, case fatality rates, and all-cause mortality. Change in population sitting time resulted in a change of disease incidence using PIFs for categorical relative risk of the five SB-related diseases [27, 62]. These epidemiological inputs were taken from the Global Burden of Disease (GBD) 2019 [63]. Morbidity impacts were quantified using years lived with disability multiplied by disease-specific disability weights from the GBD study [64].

The time spent in each health state was aggregated to estimate health adjusted life years (HALYs). Baseline sitting times were taken from National Health Survey 2014 [65]. The comparator population was identical to the intervention population except for the changes in daily sitting time. Data on healthcare costs for either incident or prevalent cases from the Australian Institute of Health and Welfare [66, 67] were inflated to 2019 prices using the Health Price Index [68, 69].

### Cost-effectiveness modelling

A substantial component of costs for intervention implementation are borne by employers. For that reason, our analyses took included a limited societal perspectives for the costing. Under a limited societal perspective, costs accrued by all the main stakeholders (both government and non-government) from different sectors (including employers, as well as individuals) were captured in the evaluation. However, due to insufficient data availability, some indirect costs such as productivity gains/losses were not included. The time horizon for the implementation of the SB interventions was one year; intervention effects were assumed to last up to 10 years after intervention (with 10% decay); the impacts of the change in SB on chronic diseases and associated health and cost outcomes were tracked over the lifetime of the modelled population. All costs and benefits were discounted using a 3% discount factor [70], and reported in 2019 values. Modelling was undertaken in Excel 2016 and second order (parameter) uncertainty analyses were undertaken applying Monte-Carlo simulation using the Excel add-in software, Ersatz version 1.35 [71]. All results are presented with 95% uncertainty intervals (UI).

Monte-Carlo simulations (based on 2000 iterations) were used to calculate the mean incremental costs and benefits of each intervention compared to the do-nothing comparator. It was assumed that there was no intervention cost incurred for the do-nothing comparator. All costs and benefits reported in Table 5 are reported as incremental costs and benefits of the intervention compared to the do nothing comparator. The incremental cost-effectiveness ratio (ICER) was calculated by dividing the incremental cost by incremental HALYs of the intervention compared to the do-nothing comparator. The intervention was judged to be cost-effective if the ICER was less than the commonly used willingness to pay threshold for Australia of A$50,000 per HALY gained [61, 72, 73]. This threshold has been used in several previous economic evaluations in the Australian context [27, 74, 75]. The results of the cost-effectiveness modelling were plotted on a cost-effectiveness plane to demonstrate the distribution of ICERs for the different interventions modelled.

### Sensitivity analyses

One-way sensitivity analyses and scenario analyses were undertaken to assess the impact of key assumptions on the cost-effectiveness results. We tested intervention uptake rates of 0.37% [76] and 2% [36]. Different intervention effect sizes (for MI and EI) reported by multiple published meta-analyses were also tested. Decay rates of 5% and 10% were modelled in combination with threshold analyses of different levels of equipment discount (30%, 50% and 70%). Summary of key sensitivity analyses are given in Table 3.

**Table 3:** Details of the scenario under sensitivity analyses conducted.

	Behavioural Intervention	Environmental Intervention	Multi-component Intervention
***Primary analyses***			
Intervention effectiveness	60.57 minutes (95% CI 34.46; 86.67)	64.05 minutes (95% CI 23.46; 104.64)	42.63 minutes (95% CI 7.63; 77.62)
Equipment cost	Full market price		
Decay rate	10% annually starting year 2		
***Scenario 1—Best intervention effectiveness (10% decay) with discount on equipment costs–societal perspective***			
Intervention effectiveness [21]	As in primary analyses	Best effect size: 96.72 minutes (95% CI 67.39; 126.05)	Best effect size: 104.38 minutes (95% CI 85.96; 122.81)
Equipment costs		Threshold analyses testing different discount rates on equipment costs	
Decay rate		10% annually starting year 2, s in primary analyses	
***Scenario 2—Best intervention effectiveness (5% decay) with 70% discount on equipment costs–societal perspective***			
Intervention effectiveness	N/A	Best effect size as in Scenario 1	Best effect size ss in Scenario 1
Equipment costs		70% discount on equipment costs	
Decay rate		5%	

Note: CI: confidence interval

## Results

### Intervention costs

When scaled up to a national level with a 0.37% organisation uptake rate [76], the interventions reached 1018 organisations, with a total of 1,619,239 employees. The number of participants that took up the BI was approximately 631,503, which translated to 4.12% of the target population (Australian adult population aged 20–65 years in 2019). For the EI, where the uptake was higher, the estimated number of participants was 922,966 participants (6.02% of the target population). Rolling out of the MI would reach 518,156 participants (3.38% of the target population).

The BI was estimated to cost A$159 million, with a per participant cost of A$251. Costs accrued by the healthcare sector (i.e. cost to Government) were substantially lower (A$30.9 million), than the cost borne by participating organisations (was A$128 million). The total cost of the EI was A$688 million, with a cost per participant of A$748. A MI was the most costly with a total intervention cost of A$438 million and per participant cost of A$853.

Costs of equipment such as the sit-stand workstations and the sit-stand platforms were the major cost drivers of the EI and MI, accounting for 95% and 74% of the total cost respectively. In general, the majority of the total intervention costs would be incurred by the organisations (businesses): 81% of total intervention costs in BI, 99% of the EI and 88% in MI. The cost to government would be minimal. Details of cost components by payers and by intervention are shown in Table 4.

**Table 4:** Intervention costs by component and payer.

Item	Cost Mean (95% uncertainty interval)		
***Overhead cost for program coordination***			
Project Manager	$129,164 ($122,804; $134,412)		
Project Officer time	$583,683 ($537,860; $621,635)		
Program web page annual maintenance cost	$6,416 ($4,847; $7,975)		
Landline phone	$3,960 (no range)		
***Other cost components***	**Behavioural intervention**	**Environmental intervention**	**Multi-component intervention**
Organization engagement	$204,423 ($113,047; $311,071)	$106,704 ($60,848; $163,384)	$204,745 ($114,576; $314,812)
Capacity building for champion	$122,675 ($99,926; $145,981)	No cost	$122,744 ($99,639; $146,308)
Counselling (groups and individual sessions; follow up phone calls)	$153.8M ($40.0M; $313.0M)	No cost	$89.9M ($27.2M; $192.1M)
Educational information and tracking material	$4.1M ($1.2M; $7.5M)	No cost	$3.4M ($1.5M; $5.7M)
Equipment	No cost	$653.1M ($403.5M; $948.3M)	$343.9M ($156.M; $579.7M)
***Total intervention cost***	**$159.0M ($42.3M; $320.9M)**	**$688.0M ($425.6M; $997.6M)**	**$438.2M ($195.94M; $756.23M)**
Total costs to businesses	$128.1M ($33.7M; $264.7M)	$687.3M ($424.9M; $996.9M)	$385.8M ($177.8M; $656.9M)
Total costs to Government	$30.9M ($7.3M; $68.7M)	$0.72M ($0.68M; $0.76M)	$52.4M ($15.5M; $111.7M)
Cost per participant	**$251 ($147; $366)**	**$748 ($703; $799)**	**$853 ($739; $1,009)**

Note: M: million; $: Australian dollar 2019

### Cost-effectiveness results

Cost-effectiveness modelling indicated that of the three interventions, the EI resulted in the most HALYs gained 919 (95% UI 657; 1,219), followed by the BI with 604 (95% UI 409; 852) HALYs gained, and the MI with 347 (95% UI 219; 505) HALYs. The EI was shown to result in the most healthcare cost saving (A$10.5M), followed by BI (A$6.9M), while the MI resulted in least healthcare cost savings (A$3.9M) (see Table 5).

**Table 5:** Costs effectiveness results in primary analyses.

	Behavioural Intervention Mean (95% uncertainty interval)	Environmental intervention Mean (95% uncertainty interval)	Multi-component intervention Mean (95% uncertainty interval)
**Incremental HALYs**	604 (409; 852)	919 (657; 1,219)	347 (219; 505)
**Total healthcare cost saving**	$6.9M ($4.9M; $9.1M)	$10.5M ($7.7M; $13.4M)	$3.9M ($2.7M; $5.5M)
**Incremental cost**	$152.1M ($35.6M; $312.7M)	$677.5M ($415.4M; $986.8M)	$434.2M ($192.M; $751.4M)
**ICER**	$251,863 ($58,375; $563,825)	$737,307 ($415,919; $1,225,364)	$1,250,426 ($514,408; $2,537,948)
**Probability of being cost-effective**	2%	0%	0%

**Note:** HALYs: Health-adjusted life years; ICER: incremental cost-effectiveness ratio.

**Fig 2:**
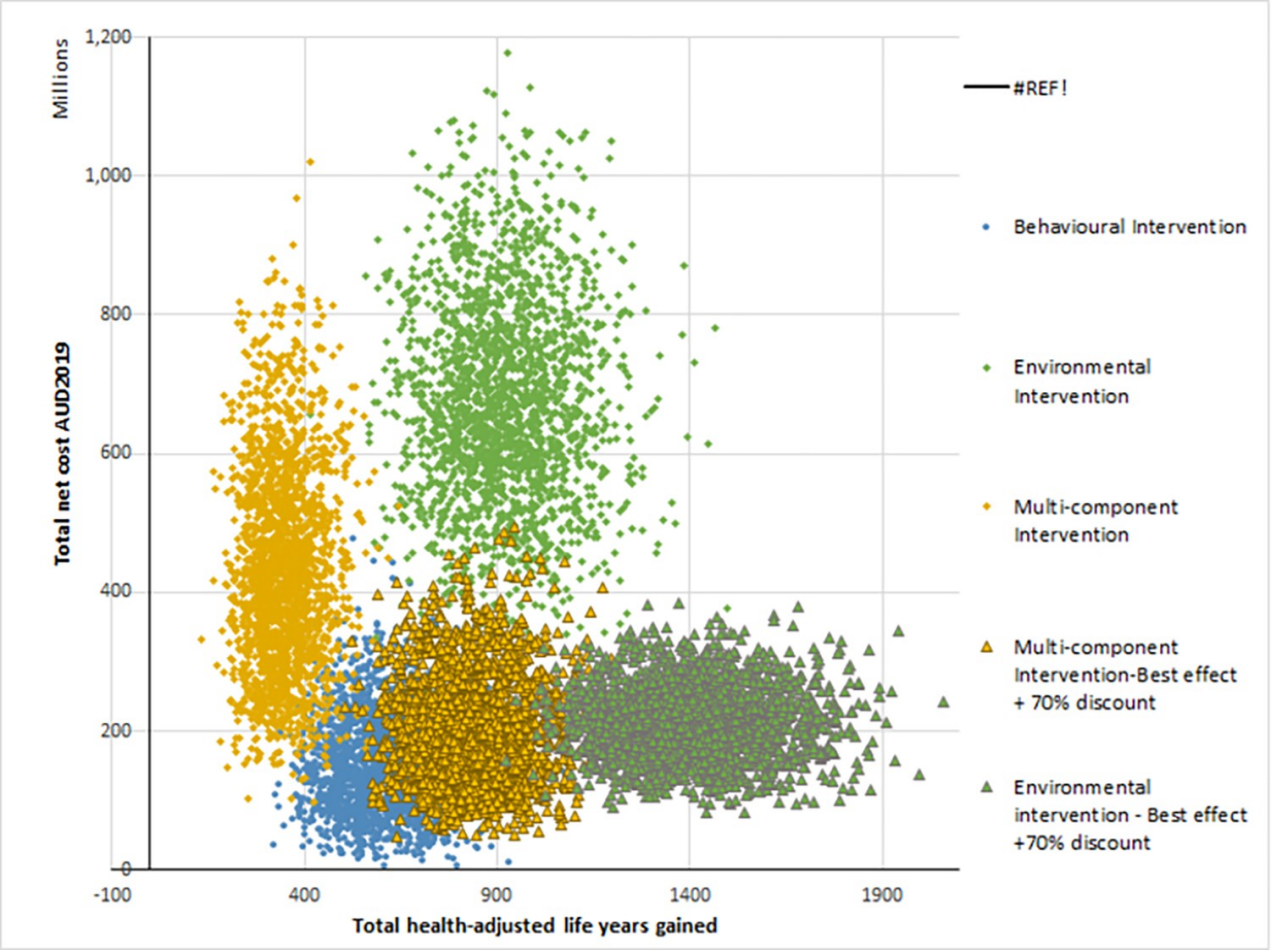
Cost-effectiveness plane.

None of the three SB interventions were cost-effective. The ICERs of the EI was A$737,307 per HALY gained, and A$1,250,426 per HALY gained for MI, both of which exceed the threshold with 0% probability of being cost-effective. The BI had an ICER of A$251,863 with only 2% probability of being cost-effective. Details of cost-effectiveness results under the primary analyses are shown in Table 5 and the cost-effectiveness plane is illustrated in Fig 2.

Under sensitivity analyses, when the best intervention effect sizes were used, the EI and MI remained not cost-effective. In scenario 1, cost of the equipment was reduced on top of best intervention effectiveness. Threshold analyses of different levels of discount on equipment costs showed an increase in the probability of the intervention being cost-effectiveness when the discounts reached 70% (Scenario 1, Table 6). At this level of discount, the average equipment cost reduced to A$251 per participant. The probability of being cost-effective was 0% under the other discount rates (30% and 50%). When the intervention decay rate was reduced from 10% to 5% per annum, the probability of the intervention being cost-effective remained low (0.6% for the environmental intervention and 0.4% for the multi-component intervention). When the organisation’s uptake rate was varied from 0.37% [76] to 2% [36], the total interventions costs increased, but the cost per participant and ICER were very similar.

**Table 6:** Cost-effectiveness results sensitivity analyses.

	Environmental Intervention Mean (95% uncertainty interval)	Multi-component Intervention Mean (95% uncertainty interval)
***Scenario 1—Best intervention effectiveness (10% decay) with 70% discount on equipment costs–societal perspective***		
Total intervention cost	$231 M ($135 M; $336 M)	$213 M ($94 M; $383 M)
Cost per participants	$251 ($237; $266)	$411 ($310; $547)
Incremental HALY	1,415 (1,108; 1,770)	845 (629; 1,087)
Total healthcare cost saving	$16 M ($13 M; $19 M)	$10 M ($7 M; $12 M)
Incremental cost	$215 M ($118 M; $320 M)	$203 M ($84 M; $372 M
ICER	$151,687 ($80,273; $245,247)	$240,174 ($95,683; $483,749)
Probability of being cost-effective	0%	0%
***Scenario 2—Best intervention effectiveness (5% decay) with 70% discount on equipment costs–societal perspective***		
Total intervention cost	As in scenario 1	As in scenario 1
Cost per participants	As in scenario 1	As in scenario 1
Incremental HALY	1,871 (1,449; 2,351)	1,131 (847; 1,475)
Total healthcare cost saving	$21,076,993 ($17,277,222; $25,345,919)	$12,732,893 ($9,884,879; $15,945,104)
Incremental cost	$210,636,698 ($118,355,565; $314,718,378)	$198,819,628 ($80,585,464; $369,676,119)
ICER	$112,574 ($58,549; $184,220)	$175,859 ($68,852; $346,183)
Probability of being cost-effective	0.6%	0.4%

Note: HALY: health-adjusted life year; ICER: incremental cost-effectiveness ratio.

As expected, the cost-effectiveness plane (Fig 2) showed that the environmental and multi-component interventions moved closer to the threshold of A$50,000 per HALY gain threshold when the effectiveness of the intervention was assumed to increase together with a reduced equipment costs. However, the interventions remained not cost-effective.

## Discussion

Our findings indicate that SB interventions are not cost-effective when reduction in sitting time is used as the risk factor for modelling. The cost-effectiveness results are heavily driven by the cost of the sit-stand desks and the small number of HALYs gained from the impact of sitting on chronic disease. Our findings indicate that MI are very costly, with the additional costs of such interventions not being justified by the resulting improvements in health outcomes. This about provided important information to guide resource allocation to reduce SB.

The findings of this current study diverge from the current evidence drawn from other economic evaluations of SB interventions. A number of factors contribute to the differences in results, in particular, the intervention costs included, health benefits gained, and the modelling approach taken. In this section, we will discuss each factor.

The cost per participant of A$853 for the MI was higher compared to Stand Up Victoria which reported a cost per participant of A$474, however the equipment costs for the latter were only A$295.70 (including delivery) in 2014. While both studies used similar costing approaches, the key cost difference was the price of the sit-stand workstation. In this analysis the 2019 market price was used, ranging from $703.00 to $960.00 per unit, while the Stand Up Victoria trial costed the same workstation at $185.70 in 2014. Other MIs included Dynamic Work (in Netherlands) with a cost of A$597 per participant [24]; and SMArtWork (in the UK): A$1283 per participant (GBP 595) [25], making the cost per participant in our study similar to the average cost of Dynamic Work and SMArtWork.

While our analysis is a model-based economic evaluation, the evaluation of Dynamic Work was a within-trial cost-effectiveness analysis with benefits reported in QALYs, measured using participant reported EQ-5D-5L. The incremental cost-effectiveness ratio for this study was reported as EUR20,000/QALY and was concluded to be cost-effective [24]. The evaluation of SMArtWork was, on the other hand, a cost-benefit analysis taking into account productivity costs (absenteeism, presenteeism and work productivity), and reported to have positive net value [25].

In the context of the long-term health benefits, our study showed that, when reduction in sitting is used as the risk factor for economic modelling, it resulted in less HALYs gained compared to the use of changes in standing time previously reported in other economic analyses [23]. For instance, when an increase in standing time of 42.2 minutes/8h day was reported and used as the physical activity risk factor in the modelling, the Stand Up Victoria intervention was reported to be cost-effective when modelled for five PA-related diseases (namely ischemic stroke, ischemic heart disease, breast cancer, colon cancer and diabetes).

Meanwhile, a similar reduction in sitting time of 42.63 minutes per day (as reported in Stand Up Victoria [23]) would not result in an ICER below the cost-effectiveness threshold when using the same model. This is primarily because the relative risks for the disease conditions associated with physical activity and SB are very different, and these are the key drivers of the long-term health benefits. For instance, the relative risks of excessive sitting and T2D and stroke in this current model were 1.31 (95% CI 1.15; 1.48) and 1.21 (95% CI 1.07; 1.37) [27] respectively; while the magnitude of these disease risks due to physical inactive in the model were 1.76 and 1.72 [77].

These findings highlight that future research should consider the development of a novel methodology to capture the health benefits of simultaneously reducing sitting time and increasing standing time for such interventions, adjusting for double counting. This would require advancements in the understanding of the relationship between SB and physical activity as risk factors for chronic illness and these relationship would need to be modelled. The current focus of our study (and that of others) on only one outcome measure suggests that the benefits of the interventions are being understated. Moreover, these cost-effectiveness results should be interpreted with appropriate caution, as our model included only five SB-related chronic diseases [27], and SB is associated with other benefits that are not included in the current model, such as metabolic, physical, mental health and musculoskeletal outcomes. The model is, therefore, likely to underestimate the potential health impacts, which has been previously discussed elsewhere [27]. As such, while we await methods to advance the capture of the joint benefits of both reduced sitting and increased standing, we recommend that interventions targeting reduction in sitting time also measure the change in PA of any level of intensity in METs rather than just an increase in standing time.

While sit-stand desks can be considered a costly item, a recent survey indicated that both workplaces and individuals are willing to invest in sit-stand workstations/desks. A survey found that 80% of organisations that participated in the BeUpstanding program had already invested in sit-stand workstations [78]. The interviews revealed that employers who had invested in sit-stand workstations perceived them to be effective in reducing discomfort and increasing employee productivity and work satisfaction [78]. Another business report of a desk supplier showed that sales of sit-stand workstations increased by 400% since the start of the coronavirus pandemic in early 2020, with 98% of the orders delivered to home addresses [79]. These statistics indicate a high willingness to pay by individuals. Given the recent changes in work environment since the start of the COVID19 pandemic, the effectiveness of SB interventions need to be further investigated.

Whilst 99% of the intervention costs were borne by industry, government involvement could result in better ability to purchase in bulk at discounted prices. Some initial work has been conducted by Australian Federal authorities and a research institute under the program to create healthy workplaces by reducing prolonged sitting time: Healthy workplace program by VicHealth [80]. Moreover, the cost of sit-stand platforms and sit-stand workstations in this analysis were for high-end equipment. Bulk buy discounts of up to 70% might not be feasible, even for large businesses, but would bring the interventions closer to being cost-effective. Small business can always select other more reasonably priced products, which can be as low as A$199 for a sit-stand platform [81]. It is also noteworthy that the equipment costs in our study were not incremental, i.e. the difference between a normal sitting desk versus a sit-stand desk; and were not annuitized over time. With modern office fit-outs, having sit-stand workstations as the default option, the incremental cost (sit-stand desk versus sitting desk) should reduce significantly.

The current evidence showed that previous interventions heavily relied on individuals to conduct the behavioural aspects of the intervention, i.e. having face-to-face behavioural change components. More recent trials have begun to utilise digital technology to implement these aspects on a wide scale [30]. In doing so, it is possible that this approach could drastically reduce the personnel costs associated with behavioural interventions, however their effectiveness has not yet been established. A digitally delivered intervention could be explored in future intervention research.

This research not only highlighted and robustly compared the cost-effectiveness of different interventions designed to reduce sitting time, but also provided insights for health policy makers within the Australian context about the value-for-money afforded by different types of SB interventions. Importantly, it took into account other important implementation considerations (strength of evidence, equity, acceptability, feasibility, sustainability and side effects) which are relevant to policymakers when making resource allocation decisions, and did not place undue reliance on the technical cost-effectiveness results. This study is not without its limitations. Firstly, SB interventions could offer additional benefits due to the improved productivity of employees (both absenteeism due to sick leave or presenteeism) which were not captured in this research. These non-health benefits should be the focus of future research as they are strongly related to the working environment and offer great incentives for employers to invest in SB intervention as part of workplace well-being programs. Secondly, the effect sizes used in primary analyses were from a systematic review undertaken by Blackburn et al [19] that included interventions outside the workplace, i.e. community, primary care with/without home setting. These results may have underestimated the effectiveness of work-place interventions. For that, we tested other effect sizes from a Cochrane systematic review with meta-analyses [21] under sensitivity analyses. Whilst we tested different scenarios, we acknowledge that other model structural issues such as other relevant health states or alternative transition probabilities were not assessed.

## Conclusion

In conclusion, of the three types of SB interventions most frequently implemented in workplaces, only BI had a small probability of being cost-effective when evaluated using a limited societal perspective. EI and MI were not cost-effective. Future research should focus on capturing non-health-benefits of these interventions, such as productivity, work satisfaction; as well as other health benefits: metabolic, physical, musculoskeletal outcomes. Importantly, for interventions that target reduced sitting time, this is replaced by other activities such as standing or walking which is associated with varied levels of PA. Although SB and PA are two distinct risk factors, the joint health benefits of simultaneously reducing sitting time and increasing standing time for such interventions should be captured, without double counting the benefits.
